# Targeting Glioblastoma Stem Cells with 2-Deoxy-D-Glucose (2-DG) Potentiates Radiation-Induced Unfolded Protein Response (UPR)

**DOI:** 10.3390/cancers11020159

**Published:** 2019-01-31

**Authors:** Sumedh S. Shah, Gregor A. Rodriguez, Alexis Musick, Winston M. Walters, Nicolas de Cordoba, Eric Barbarite, Megan M. Marlow, Brian Marples, Jeffrey S. Prince, Ricardo J. Komotar, Steven Vanni, Regina M. Graham

**Affiliations:** 1 University of Miami Brain Tumor Initiative, University of Miami Miller School of Medicine, Miami, FL 33136, USA; sumedhss@med.miami.edu (S.S.S.); gregor.rodriguez@med.miami.edu (G.A.R.); WWalters@med.miami.edu (W.M.W.); nicolas.a.decordoba@email.shc.edu (N.d.C.); eric_barbarite@meei.harvard.edu (E.B.); mmarlow135@yahoo.com (M.M.M.); brian.marples@med.miami.edu (B.M.); RKomotar@med.miami.edu (R.J.K.); 2 Department of Neurological Surgery, University of Miami Miller School of Medicine, Miami, FL 33136, USA; SVanni@med.miami.edu; 3 Dauer Electron Microscopy Laboratory, Department of Biology, University of Miami, Coral Gables, FL 33146, USA; am722@duke.edu (A.M.); jeffprince@miami.edu (J.S.P.); 4 Department of Radiation Oncology, University of Miami Miller School of Medicine, Miami, FL 33136, USA; 5 Sylvester Comprehensive Cancer Center, University of Miami Health System, Miami, FL 33136, USA

**Keywords:** cancer stem cells, ER stress, glioblastoma multiforme, radiation, unfolded protein response, autophagy

## Abstract

Glioblastoma (GBM) is the most common and aggressive primary brain tumor in adults, and despite optimized treatment options, median survival remains dismal. Contemporary evidence suggests disease recurrence results from expansion of a robustly radioresistant subset of GBM progenitor cells, termed GBM stem cells (GSCs). In this study, we utilized transmission electron microscopy to uncover ultrastructural effects on patient-derived GSC lines exposed to supratherapeutic radiotherapy levels. Elevated autophagosome formation and increased endoplasmic reticulum (ER) internal diameter, a surrogate for ER stress and activation of unfolded protein response (UPR), was uncovered. These observations were confirmed via protein expression through Western blot. Upon interrogating genomic data from an open-access GBM patient database, overexpression of UPR-related chaperone protein genes was inversely correlated with patient survival. This indicated controlled UPR may play a role in promoting radioresistance. To determine if potentiating UPR further can induce apoptosis, we exposed GSCs to radiation with an ER stress-inducing drug, 2-deoxy-D-glucose (2-DG), and found dose-dependent decreases in viability and increased apoptotic marker expression. Taken together, our results indicate GSC radioresistance is, in part, achieved by overexpression and overactivation of ER stress-related pathways, and this effect can be overcome via potentiation of UPR, leading to loss of GSC viability.

## 1. Introduction

Glioblastoma (GBM) is the most common and aggressive form of primary brain cancer, and despite optimized therapy consisting of surgery, chemotherapy, and radiation, its expected median survival remains under two years [1]. Informed by techniques used to enrich and characterize neural stem cells, several groups have demonstrated GBM, along with other primary brain tumors, contains self-renewing, tumorigenic cells referred to as tumor initiating cells, GBM stem-like cells, or GBM stem cells (GSCs) [2,3,4,5]. GSCs are implicated in tumor recurrence through mechanisms including expression of drug efflux pumps and ability to withstand radiotherapy, and thus, characterizing resistance mechanisms may uncover potential therapeutic targets and augment current cancer therapy [6].

Within the tumor microenvironment, a multitude of metabolic stresses (i.e., hypoxia, nutrient depletion, etc.) results in selective pressure on GSCs [7,8,9]. Activation of pro-survival pathways designed to mitigate the effect of these stressors can inadvertently impart a survival advantage to cells exposed to cytotoxic therapy. One such survival mechanism is stimulation of the endoplasmic reticulum (ER) stress pathway, commonly referred to as the unfolded protein response (UPR) [10,11,12,13]. Unfolded proteins accumulate in the ER secondary to energy depletion and/or environmental stresses, leading to the activation of PERK, IRE1, and ATF6—transducers of UPR that reestablish ER homeostasis via ER-associated protein degradation, translation of protein folding chaperones, and attenuation of global protein synthesis [14]. Activation of the ER stress pathway can also induce autophagy, which can help strengthen resistance to cellular stresses [15]. Ultimately, if ER homeostasis cannot be achieved, then UPR navigates the cell towards apoptosis.

One of the many critical downstream effects of UPR is the upregulation of glucose related proteins 78 (GRP78) and 94 (GRP94), multifunctional members of the heat shock protein family [16]. GRP78 acts to regulate PERK, IRE1, and ATF6 activation, while both GRP78 and GRP94 are protein-folding chaperones [17,18]. Elevated baseline GRP78 expression is also related to increased malignancy and radiotherapy resistance in multiple cancers [19,20,21,22]. In GBM, Dadey and colleagues showed that radiation potently activates UPR and subsequently upregulates GRP78, and antibody targeting of GRP78 in non-stem GBM cells enhanced efficacy of radiation [23,24]. Targeting UPR using the ER-stress potentiating drug, celecoxib, also sensitized GBM cells to radiotherapy [25].

Despite emerging evidence that ER-stress potentiation may be a viable therapeutic option in cancer treatment, exploitation of this pathway in cancer stem cells—notable for their robust radioresistance—remains understudied. In this present study, we exposed patient-derived GSC lines to radiotherapy and the ER-stress inducing agent, 2-deoxy-D-glucose (2-DG), to test our hypothesis that potentiating radiation-induced ER-stress in the GSC population leads to decreased cell viability. Our confirmation of this hypothesis reveals a novel understanding of a pathway GSCs utilize to evade radiation cytotoxicity and suggests a possible therapeutic target to circumvent this resistance.

## 2. Results

### 2.1. GSCs Exhibit Robust Radioresistance

**Figure 1:**
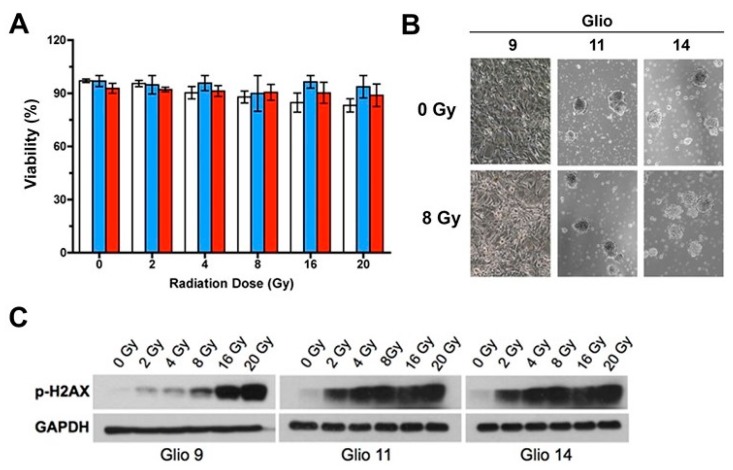
Glioblastoma stem cells (GSCs) exhibit robust radioresistance. (**A**) Viability as determined by trypan blue exclusion assay of Glio9 (white), Glio11 (blue), and Glio14 (red) treated with increasing doses of radiation and then analyzed 72 h after exposure. Results are representative of at least three experiments and displayed as mean ± SEM. (**B**) Light microscopy images of GSC neurosphere phenotype with and without radiation treatment. Magnification: 10×. (**C**) Western blot of p-H2AX, a marker for double strand breaks, in GSCs exposed to increasing radiation.

To characterize GSC radioresistance, we exposed three established GSC lines—Glio9, Glio11, and Glio14—to increasing levels of ionized radiation (one-time dose: 0 Gy, 2 Gy, 4 Gy, 8 Gy, 16 Gy, and 20 Gy) and determined the cell viability after 48 h using a trypan blue exclusion assay. We observed a negligible decrease in cell viability despite administering supratherapeutic levels of radiation (Figure 1A). Utilizing light microscopy, we qualitatively analyzed GSC morphology 48 h post irradiation with 8 Gy and found no ultrastructural aberrations secondary to radiation treatment (Figure 1B). GSCs grown in normal culture conditions maintain their phenotype as spheroid clusters of cells, termed neurospheres, and a morphology change towards a monolayer pattern indicates loss of stemness [2]. Therefore, we found radiation did not negatively affect either stem phenotype or GSC viability.

To confirm that radiation induced cellular damage in our GSC lines, we examined H2AX phosphorylation 1h post radiation exposure. As a variant member of the H2A histone family, H2AX phosphorylation is the first step in recruiting and localizing DNA repair proteins, so it is often used as a marker for double-stranded breaks [26]. We found radiation induced DNA damage in treated cell lines; however, no identifiable loss of cell viability or form follows (Figure 1C). These data suggest that our patient-derived GSC lines are resistant to supratherapeutic levels of radiation despite accumulation of DNA insults.

### 2.2. Radiation Exposure Induces Autophagosome Formation and Activates UPR in GSCs

**Figure 2:**
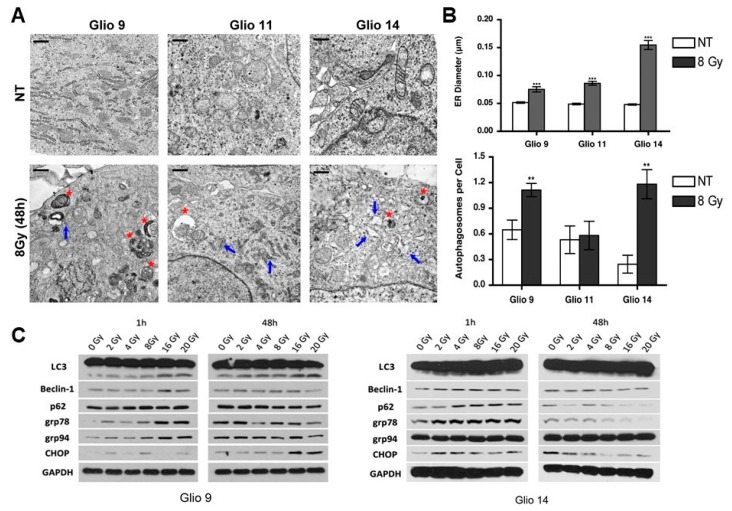
Radiation induces autophagosome formation and activation of unfolded protein response (UPR) in GSCs. (**A**) Transmission electron microscopy (TEM) analysis of Glio9, Glio11, and Glio14 comparing ultrastructural responses in non-treated lines and cells exposed to 8 Gy of radiation. Endoplasmic reticulum (ER, blue arrows) and autophagosomes (red asterisks) are highlighted. Five images from two independent studies were analyzed. Scale bar: 0.5 µm. (**B**) Quantitative analysis of ER luminal diameter, as a surrogate marker for ER stress, and autophagic vesicles per cell using TEM images. Results are representative averages of at least five images and are displayed as mean ± SEM. ** *p* < 0.01, *** *p* < 0.001. Mann-Whitney test. (**C**) Western blot analysis for ER stress markers (GRP78, GRP94, CHOP) and autophagy markers (LC3, Beclin-1, p62) in Glio9 and Glio14 at 1 h and 48 h post radiation exposure to increasing doses. See also Figure S1.

After observing GSCs are highly radioresistant despite accumulation of significant DNA damage, we studied post-radiation cellular modifications using transmission electron microscopy (TEM) to elucidate potential survival strategies GSCs employ to circumvent radiation-mediated cell death. GSCs were treated with a single dose of 8 Gy radiation and were prepared for TEM after 48 h to allow sufficient time to capture any morphological changes. Compared to NT, radiotherapy treated lines exhibited increased ER luminal diameter (blue arrows) and higher number of autophagic vesicles (red asterisk) (Figure 2A,B). Dilation of the ER has been used as a marker for ER stress in cancer [27,28], thus our observations confirmed radiation induced ER stress. Table 1 shows ER diameter in microns and autophagic vesicles per cell between cell lines and treatment cohorts. Of note, while all GSCs lines exhibited increased stress responses, only Glio11 did not present with significantly higher autophagosome formation in NT versus radiotherapy treated cells. Consistent with these findings, inhibition of autophagy with chloroquine prior to radiotherapy decreased cell number compared to radiotherapy alone in both Glio9 and Glio14 but not in Glio11 suggesting that autophagy does not play a major role in mediating radioresistance in Glio11 cells (Figure S1).

After observing morphological changes using TEM, we performed western blot analysis (Figure 2C) for markers of UPR (GRP79, GRP94, and CCAAT-enhancer-binding protein homologous protein (CHOP)) and autophagy (LC3, Beclin1, and p62) at early (1 h) and late (48 h) timepoints after exposure to increasing doses of radiation. By 1 h post exposure, we see a dose dependent activation of stress factors like the GRPs; however, CHOP activation, a potent mediator of UPR-associated apoptosis did not follow identifiable trends. For autophagy-related protein products, we observed a dose-dependent increase in all targets probed. At 48 h, most effects seen at 1h plateaued (as in the case of Beclin1, p62, GRP94) or began returning to NT baseline (with LC3, GRP78, CHOP). Taken together, our results show that radiation rapidly induces stress adaptive mechanisms, such as UPR and autophagy, and these effects can persist 48 h after single dose.

### 2.3. Upregulation of UPR Genes in Human GBM Specimen Correlates with Reduced Patient Survival

**Figure 3:**
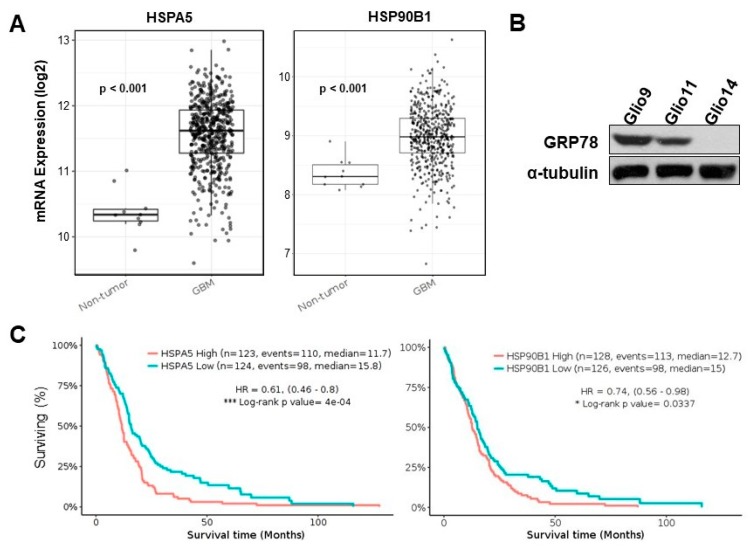
Upregulation of UPR genes in human GBM specimen correlates with reduced patient survival. (**A**) Comparison of non-tumor (*n* = 10) and GBM sample (*n* = 528) for mRNA expression of ER stress genes *HSPA5* and *HSP90B1*. Pairwise *t*-test. (**B**) Western blot analysis for baseline levels of GRP78 in Glio9, Glio11, and Glio14. (**C**) Kaplan-Meier plot of survival using the GlioVis portal comparing low expression (25th percentile) and high expression (75th percentile) of *HSPA5* and *HSP90B1*. Results reported as hazard ratio (95% confidence interval). * *p* < 0.05, *** *p* < 0.001. Log-rank test. Events = number of patients who died. See also Figure S2.

Overexpression of the UPR genes that encode for GRP78 and GRP94 have been linked to radioresistance and in multiple cancer types, including breast, gastric, and pancreatic cancers [29,30,31]. We interrogated the TCGA database via the open-access analysis platform, GlioVis, to determine if upregulation of GRP78 and GRP94 is observed in GBM patients compared to non-tumor controls and if higher expression is clinically relevant to patient survival. Genomic data from the Human Genome U133 (HG-U133) array was deciphered. Comparisons were between the 75th percentile of expression vs. the 25th percentile (high vs. low expression). We found that GBMs overall exhibit increased GRP78 and GRP94 expression compared to non-tumor controls (Figure 3A).

mRNA Log2 expression comparisons between non-tumor control and GBM specimen, respectively, were as follows: *HSPA5*, 10.38 ± 0.01 vs. 11.56 ± 0.02, *p* < 0.001; *HSP90B1*, 8.36 ± 0.08 vs. 8.98 ± 0.02, *p* < 0.001. From Western blots of our three patient samples, we noted heterogenous expression of GRP78; Glio9 displayed the highest level of baseline GRP78, followed by Glio11, and then Glio14 (Figure 3B). Should GRP78 expression be related to therapy resistance, we predicted that Glio9 would exhibit the most resistance to ER stress inducing stimuli. Interestingly, Glio9 was derived from a patient with a recurrent tumor. Finally, we found that higher vs. lower expression is correlated with significant differences in patient survival for both GRP78 and GRP94 (Figure 3C); Hazard ratio 0.61 (0.46–0.8) and 0.74 (0.56–0.98), respectively). Median survival time for high vs. low GRP78 and 94 expressions were 11.7 vs. 15.8 months, *p* < 0.0001, and 12.7 vs. 15.0 months, *p* = 0.0337, respectively.

We then decided to determine if differential autophagy gene expressions independently correlated with patient survival and found that the genes encoding for LC3, Beclin1, p62, ATG5, ATG7, and ATG12 were not correlated with patient survival (Figure S2). Additionally, the differences in non-tumor vs. GBM mRNA expression of the autophagy products were not as pronounced as those of UPR-related genes. These results indicate that upregulation of UPR genes, but not of autophagy genes, is clinically relevant and related to worse patient outcome.

### 2.4. 2-DG Induces ER Stress in GSCs in a Dose-Dependent Manner

While UPR attempts to reestablish ER homeostasis by managing accumulation of unfolded proteins, the outcome if homeostasis cannot be achieved is cell death [14]. We hypothesized that the addition of a supplementary ER stress source could potentiate the effects of radiation-induced UPR and force GSCs towards apoptosis. We, therefore, looked to the glucose analog, 2-DG, to induce ER stress in GSCs with the intention of combining it with radiation.

**Figure 4:**
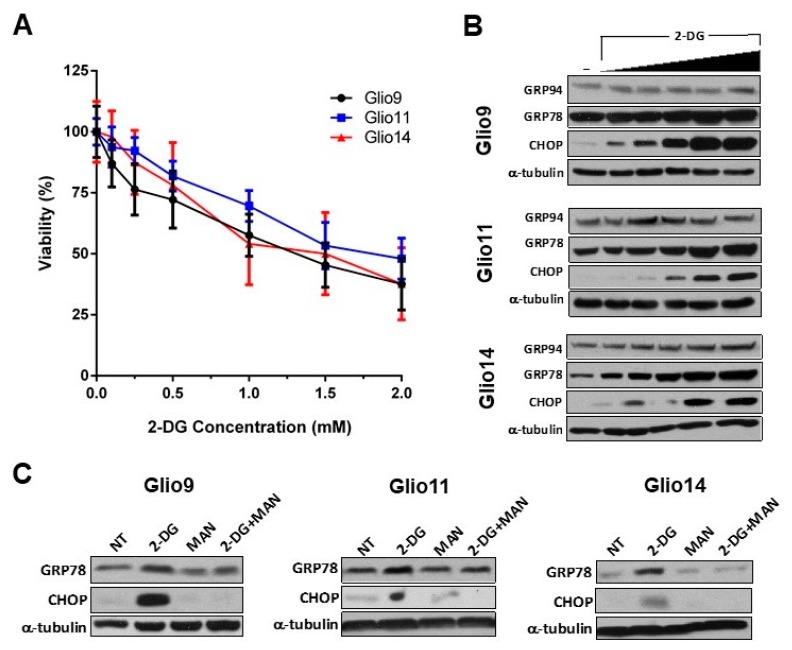
2-Deoxy-D-glucose (2-DG) induces ER stress in GSCs in a dose-dependent manner. (**A**) CellTiter 96^®^ AQ_ueous_ One Solution Cell Proliferation (Promega, Madison, WI, USA) assay of Glio9, Glio11, and Glio14 treated with increasing doses of 2-DG for 72 h. Viability is reported as mean ± SEM. Data represent average values of at least three independent experiments. (**B**) Western blot analysis for ER stress markers (GRP78, GRP94, and CHOP) in GSC lines treated with 0.0, 0.25, 0.5, 1.0, 2.0, and 4.0 mM of 2-DG for 24 h. (**C**) Western blot analysis of GSCs treated with 2-DG and/or mannose to determine mechanism of rescue from ER stress.

2-DG induces ER stress in multiple carcinoma types and has been used in combination with metformin to inhibit invasiveness and stem phenotype of GBM [32,33]. In a dose-escalation trial, we treated all our GSC lines with increasing concentrations of 2-DG (0.0, 0.1, 0.25, 0.5, 1.0, 1.5, and 2.0 mM 2-DG) and determined viability via MTS assay. A recent Phase I trial studying 2-DG combination with docetaxel in patients with advanced solid tumors found that peak plasma levels of 2-DG reach a median of 116 µg/mL, or 0.7 mM [34]. Therefore, utilizing concentrations from 0.0–2.0 mM is clinically relevant. Our viability assay revealed a modest, but significant, dose-dependent decrease in GSC viability with increasing 2-DG dose (Figure 4A). IC_50_ were determined using the calculated regression trendline for each cell line: Glio9, 1.38 ± 0.134 mM; Glio11, 1.77 ± 0.130 mM; Glio14, 1.46 ± 0.366 mM.

To confirm that 2-DG induced dose-dependent ER stress and activated UPR, we used western blot analysis of GRP78, GRP94, and CHOP in GSC lines treated with 0.0, 0.25, 0.5, 1.0, 2.0, and 4.0 mM 2-DG for 24 h. Similar to the effects of radiation, we see that UPR-associated products are upregulated in the presence of 2-DG; however, CHOP, a potent activator of UPR-mediated apoptosis, is also upregulated—a finding not seen in radiation-induced UPR (Figure 4B). Nonetheless, the viability of cells treated with achievable plasma 2-DG concentrations (0.7 mM) only is modest.

We next sought to elucidate the mechanism of ER stress induction via 2-DG in GSCs. While 2-DG is a glycolytic inhibitor [35], Kovács et al. found in endothelial cells that 2-DG interferes with N-linked glycosylation and cotreatment with 1 mM mannose can rescue cells [36]. GSC treatment using 2 mM 2-DG upregulates GRP78 and induces apoptosis via CHOP, but, similarly to the results of Kovács and colleagues, cotreatment with 1 mM mannose reverses these effects (Figure 4C). Taken together, we observed 2-DG induces UPR and apoptosis at clinically achievable concentrations, and that this ER stress is, in part, mediated by N-linked glycosylation since cotreatment with mannose prevents increased activation of UPR.

### 2.5. Combination Radiotherapy and 2-DG Increases ER Dilation in GSCs

**Figure 5:**
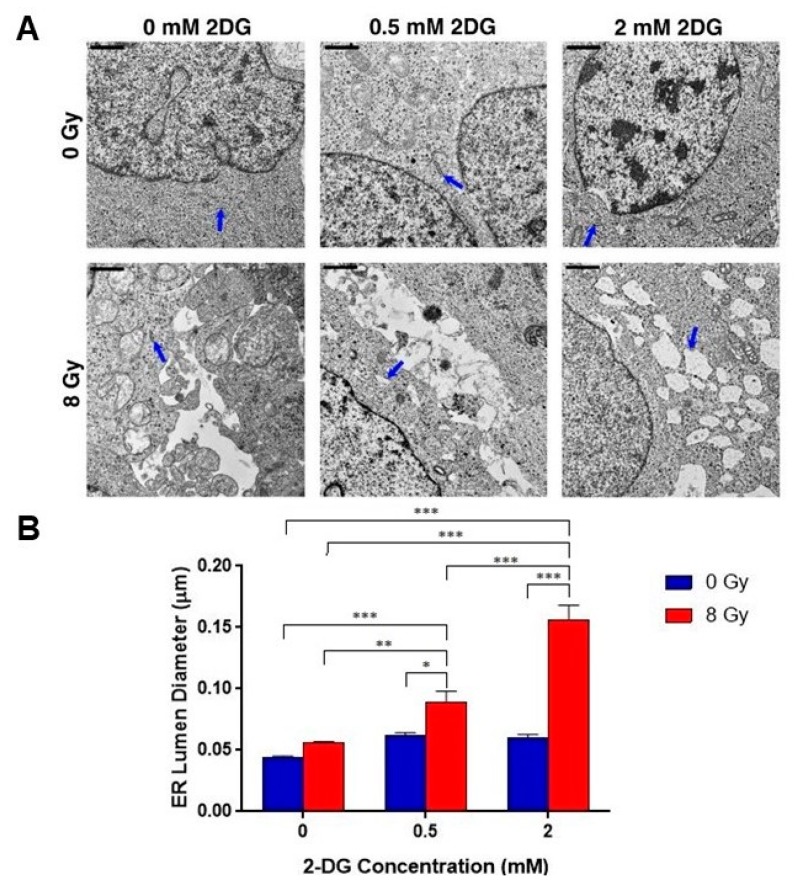
Combination radiotherapy and 2-DG increases ER dilation in GSCs. (**A**) Transmission electron microscopy (TEM) analysis of GSCs (Glio14 shown) comparing ultrastructural responses in non-treated lines and cells exposed to 8 Gy of radiation and/or increasing concentrations of 2-DG. Endoplasmic reticulum (ER) highlighted with blue arrows. Scale bar: 0.5 µm. (**B**) Quantitative analysis of ER luminal diameter, as a surrogate marker for ER stress, using TEM images (Glio14 shown). Results are representative averages of at least five images from two independent studies and are displayed as mean ± SEM. * *p* < 0.05, ** *p* < 0.01, *** *p* < 0.001. Mann-Whitney test.

Given our results that 2-DG induces ER stress in GSCs, we revisited TEM analysis to determine if combination therapy with radiation and 2-DG can induce greater ER dilation, and therefore ER stress, than either condition independently. Glio14 was treated with a single dose of 8 Gy radiation and incubated with either 0.5 mM or 2.0 mM 2-DG for 24 h, after which electron micrographs were taken and analyzed for ER dilation using ImageJ (Figure 5A). Qualitatively, we observed increased ER diameter in the dual-treated conditions than in radiation or 2-DG alone, 0.5 mM and 2.0 mM. When we quantitatively analyzed the data, we found statistically significant increases in ER diameter when dual treatment was compared to single therapy (Figure 5B). Refer to Table 2 for average diameter in microns with SEM. With this result, we see that ER stress can be potentiated by combination therapy of two ER stress inducing stimuli, and that there is an identifiable morphological consequence in GSCs.

### 2.6. Radiotherapy and 2-DG Cotreatment Potentiates UPR and Leads to Dose-Dependent Loss of Cell Viability and Apoptosis

**Figure 6:**
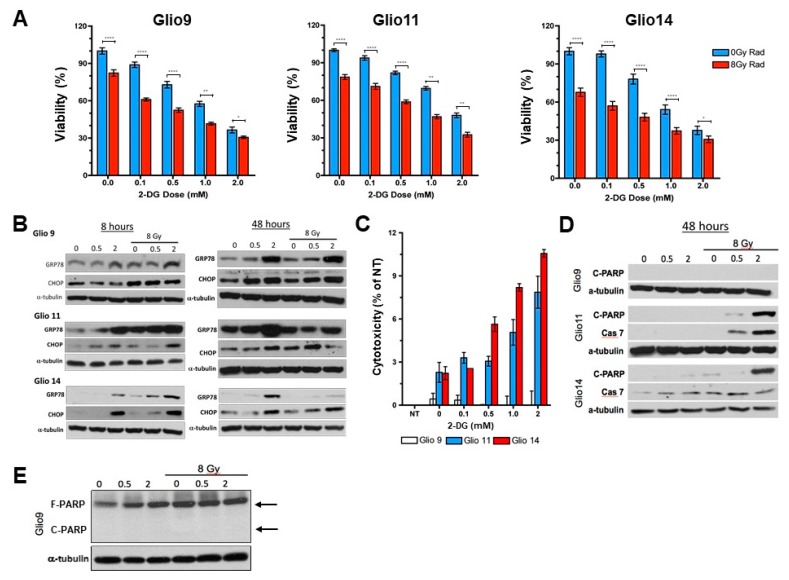
Radiotherapy and 2-DG cotreatment potentiates UPR and leads to dose-dependent loss of cell viability and apoptosis. (**A**) Proliferation assay of Glio9, Glio11, and Glio14 treated with increasing doses of 2-DG with or without 8 Gy radiation for 72 h. Viability is reported as mean ± SEM. Data represent average values of at least three independent experiments. * *p* < 0.05, ** *p* < 0.01, *** *p* < 0.001. Mann-Whitney Test. (**B**) Western blot analysis for ER stress markers GRP78 and CCAAT-enhancer-binding-protein homologous protein (CHOP) after treatment using increasing doses of 2-DG with or without 8 Gy radiation. Cells were analyzed at early (8 h) and late (48 h) time points. (**C**) Lactate dehydrogenase assay to determine cytotoxicity of Glio9 (white), Glio11 (blue), and Glio14 (red) treated with 2-DG. Cytotoxicity is reported as mean ± SEM. Data represent average values of at least three independent experiments. (**D**) Western blot analysis of apoptosis markers, cleaved-PARP and caspase-7, after 48 h treatment of GSCs using 2-DG with or without 8 Gy radiation. (**E**) Western blot of full-PARP and cleaved-PARP in Glio9 to confirm presence of full PARP and lack of PARP cleavage despite combination therapy.

To confirm our TEM observations that combination of multiple ER stress inducing stimuli led to increased ER stress, we first performed an MTS assay to determine viability of GSCs treated with radiation (single dose 8 Gy) and increasing concentrations of 2-DG. We found a significant decrease in viability between 2-DG treated cells versus 2-DG plus radiation for each concentration tested in all cell lines (Figure 6A). The IC_50_ of 2-DG for each cell line was also lower with 2-DG plus radiation compared to 2-DG alone: Glio9 IC_50_, 0.86 ± 0.147 mM vs. 1.38 ± 0.134 mM, *p* < 0.01; Glio11 IC_50_, 1.03 ± 0.206 mM vs. 1.77 ± 0.130 mM, *p* < 0.01; Glio14 IC_50_, 0.57 ± 0.159 mM vs. 1.46 ± 0.366 mM, *p* = 0.028. We then performed Western blot analysis (Figure 6B) to see if combination therapy upregulated UPR associated proteins, GRP78 and CHOP, more than 2-DG (0.5 mM and 2 mM) or radiotherapy alone. A short timepoint (8 h) and long timepoint (48 h) were chosen to determine if UPR activation persists. Not only is GRP78 upregulation highest with 2 mM 2-DG plus 8 Gy radiation at the short timepoint, but we see an increased CHOP upregulation as well, indicating that cells are entering apoptosis. The only cell line that showed undiscernible CHOP activity with combination therapy compared to single therapy was Glio9, indicating that potentiating ER stress can effectively induce apoptosis in cells with lower baseline GRP78 levels but possibly not in cells with higher baseline GRP78 levels (refer to Figure 3B). At 48 post radiation, GRP78 remained upregulated, but a reduction in CHOP expression was seen in Glio11 and Glio14 when compared to 2-DG only treatment. An LDH assay performed confirmed that Glio9 does not experience apoptosis because of ER stress therapy, but that Glio11 and Glio14 do (Figure 6C). To further characterize activation of apoptotic pathways in 2-DG plus radiation treated cells, we performed Western blot for cleaved PARP and upregulation of Caspase 7 (Figure 6D). As expected, Glio9 did not experience either increased cleaved PARP or Caspase 7 (despite strong full PARP expression (Figure 6E), indicating that PARP is present, but is not cleaved to activate apoptosis.

These results suggest that combination therapy with multiple ER stress inducing stimuli activates, maintains activation of, and potentiates UPR in GSCs. We see an additive effect of 2-DG on radiotherapy. While this combination strategy is effective in reducing cell viability in GSCs, cells with higher baseline GRP78 expression like Glio9 do not respond with cell death when exposed to multiple ER stressors. Ultimately, there is a potential role in ER stress inducing combination therapy in well-selected patients who are found to have normal UPR-associated gene expression since conventional radiotherapy, and some chemotherapies, induce ER stress.

## 3. Discussion

**Figure 7:**
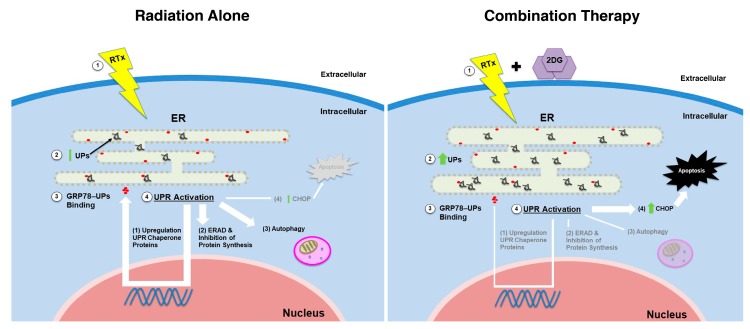
Graphic depiction of intracellular unfolded protein (UPR) activation induced by radiation versus radiation plus 2-deoxy-D-glucose (2-DG) on GSCs. **Left panel**: (1) Radiation induces accumulation of (2) unfolded proteins (UPs) in the endoplasmic reticulum (ER). This leads to release of the folding chaperone protein, GRP78, from PERK, IRE1, and ATF6 found on the ER membrane and (3) binding of GRP78 to UPs. GRP78 release activates PERK, IRE1, and ATF6 and (4) promotes UPR. Effects of activation include increased expression of chaperone proteins, ER associated protein degradation (ERAD), and autophagy, but limited apoptosis via CHOP upregulation. **Right panel**: (1) Radiation and 2-DG induce increased levels of ER stress and (2) higher levels of UPs. GRP78 binding to UPs and UPR activation follows (3,4); however, combination therapy induces higher ER stress than radiation alone, which prompt GSCs towards CHOP-associated apoptosis. Abbreviations: 2-DG, 2-deoxy-d-glucose; ER, endoplasmic reticulum; ERAD, ER-associated degradation; RTx, radiation; UP, unfolded proteins; UPR, unfolded protein response.

Despite advances in GBM therapy, overall patient survival remains dismal. A growing body of literature has focused on the role of GSCs in tumor formation, progression, and recurrence given their robust resistance to conventional chemotherapy and radiotherapy; thus, understanding mechanisms of resistance and developing cell specific therapies against GSCs are crucial in eliminating GBM [37,38]. Here, we demonstrated that radioresistance in GSCs is, in part, driven by increased expression of UPR-associated proteins, GRP78 and GRP94. However, by pharmacologically potentiating radiation-induced ER stress with 2-DG, GSC viability can be negatively affected through conversion of UPR towards CHOP-mediated apoptosis (Figure 7).

Adaptive mechanisms against the prolonged metabolic stresses in tumor microenvironment, constitutive activation of UPR, and overexpression of ER chaperone proteins like GRP78, have been studied in multiple cancer types as being linked to tumor processes such as invasion, resistance to environmental stressors, angiogenesis, oncogenic signaling, and resistance to traditional therapy [17,39]. However, should ER stress be too severe, the pro-survival function of UPR switches towards a cytotoxic pathway, leading to cell death. In this regard, targeting ER stress has emerged as a valuable mark [40].

Autophagy can be induced dependent or independent of UPR, and while we observed increased autophagosome formation in Glio9 and Glio14, we did not find increased vesicle formation in Glio11 (Figure 2A,B). When we utilized radiation and the autophagic inhibitor, chloroquine, we only observed loss of cell viability in Glio9 and Glio14. Recently, Ye et al., used chloroquine to radiosensitize glioma initiating cells (GICs); however, our results suggest that this modality is not effective in GBM cells that do not upregulate autophagy as a resistance mechanism, as is the case in Glio11 [41]. In a Phase I/II clinical trial conducted by Rosenfeld and colleagues, patients with newly diagnosed GBM were given hydroxychloroquine, a derivative of chloroquine, in addition to temozolomide and radiation. They concluded that despite hydroxychloroquine doses of 600 mg/day, consistent autophagy inhibition was not achieved and no significant improvement in overall survival was observed [42]. These findings in combination with our own suggest that GSCs are variable in their response to radiation and therapeutic use of autophagic inhibition may not be effective for all GBM patients.

Investigation into the feasibility of targeting ER stress as adjuvant to radiation therapy has proved successful. Dadey et al. concluded that induction of ER stress signaling by radiation in GBM contributes to the adaptive mechanisms encountered during radiotherapy, and that the PERK/ATF4 axis plays a critical role in maintaining viability of irradiated GBM cells [43]. Furthermore, it was also shown that antibody targeting of GRP78 attenuated cell proliferation, colony formation, PI3K/Akt/mTOR signaling, and enhanced cell death, indicating a therapeutic potential to targeting both mediators of UPR and UPR itself [24]. Pharmacological induction of ER stress has also been linked to favorable affects against GBM. Combination of 2-DG, also a potent activator of ER stress, and metformin suppressed neurosphere formation, expression of stem-cell related gene products, and invasive capacity [33]. In another study, the cyclooxygenase inhibitor, celecoxib, was utilized to enhance radiosensitivity in GBM cells through induction of ER stress [25].

Despite the expanding literature studying ER stress as a therapeutic target in GBM, the role of potentiating UPR in GBM stem cells to force cells towards CHOP-mediated cell death is still relatively novel. To our knowledge, this report represents a unique investigation seeking to characterize UPR as a radioresistance mechanism in specifically GSCs, correlate upregulation of UPR genes to overall patient survival, and propose a treatment strategy of potentiating ER stress to combat GSCs. We found evidence in The Cancer Genome Atlas (TCGA) that upregulation of UPR-associated genes is clinically relevant: patients with high expression of both *HSPA5* and *HSP90B1* exhibit decreased survival time when compared to lower expression levels (Figure 3). This data supplemented our microscopic analysis after we found a robust UPR response post radiation exposure with increased ER luminal diameter and increased autophagic vesicles—a response confirmed via Western blot. Of note, while increased autophagic vesicles were counted, autophagy related genes did not seem to be associated with survival differences in patients (Figure S1). Low levels of radiation did not induce higher CHOP expression, indicating that apoptosis may not be achieved in GSCs treated with clinical doses of radiation. We then opted to potentiate ER stress using a known ER stress inducing drug, 2-DG. In a recent phase I dose-escalation trial of 2-DG in advanced solid tumors, 2-DG was safely implemented at 63 mg/kg/day with plasma levels ~0.7 mM [34]. Combination therapy with 2-DG and radiation significantly reduced the IC_50_ of GSCs when compared to 2-DG alone, and combination therapy was responsible for increased CHOP-induced apoptosis in Glio11 and Glio14. However, this effect was not seen in Glio9, as confirmed by an LDH assay (Figure 6C).

An explanation for this lies in the variable expression of UPR protein expression at baseline (Figure 3B), with Glio9 having the highest expression, followed by Glio11, and then Glio14. While the genetic and phenotypic heterogeneity of GBM is well characterized, this result was particularly interesting since Glio9 was derived from a patient with recurrent GBM who had previously undergone the Stupp protocol (combination temozolomide with fractionated radiation) [1], as opposed to Glio11 and Glio14, which came from treatment-naïve patients. This suggests that higher levels of GRP78 expression confer a higher tolerance for ER stress inducing stimuli. As comparison, Glio14 had the lowest baseline GRP78 expression but had the highest evidence of combination therapy cytotoxicity per LDH assay. Despite a difference in cell death between cell lines, overall, GSC viability is negatively affected after treatment with combination therapy.

These results are relevant and novel for a few reasons. We showed that baseline levels of GRP78 are important in conferring resistance to radiation with or without ER stress inducing concurrent therapy. The fact that our highest GRP78 expressing cell line comes from a patient with recurrent disease also implies that GSCs with prior radiation exposure can maintain radioresistance. As such there may be a role for histologically examining GBM samples for UPR-associated protein expression prior to initiation of radiotherapy in primary or recurrent tumors, though this nascent idea would benefit from further clinically-oriented study. Our results also suggested that despite GRP78 expression, radiation plus 2-DG reduced viability of GSCs when compared to treatment with radiation alone. These results echo those achieved by several groups who used various ER stress-inducing stimuli to radiosensitize tumor cells [25,44,45,46]. Without radiation combination, the IC_50_ of GSCs exposed to 2-DG alone ranged between 1.38–1.77 mM—higher than what seems to be achievable in plasma. Combination therapy reduced the IC_50_ of all GSC lines collectively by roughly half (range, 0.57–1.03 mM).

Recently, 2-DG and radiotherapy was safely used in clinical trial against GBM in India, and trial results from over 100 patients revealed a modest survival advantage with improvement in patient quality of life [47,48]. Given intertumor variability though, future investigation would need to identify ways to increase the cytotoxicity of radiation and determine which patients would benefit most from this combination therapy. Our results suggest that examining ER-stress pathway protein expression could eventually be used for patient selection and that exploiting UPR-related apoptosis could enhance the degree of radiosensitization in GBM by targeting GSCs. Ultimately, further study is warranted via larger, prospectively-designed trials looking at multimodal potentiation of ER stress in patients selected based on UPR gene expression on histology.

## 4. Materials and Methods

### 4.1. Tissue Culture and Reagents

Collection of patient-derived GBM samples for use in biomedical research was approved by the Institutional Review Board (6 June 2007) at the University of Miami in accordance to the Declaration of Helsinki. Prior to harvesting of GBM specimen, informed consent was obtained from all patients by the attending Neurosurgeon, a clinical research coordinator, or a trained pathology assistant.

With Institutional Review Board (IRB) at the University of Miami approval and patient written informed consent, three GBM tumor samples were harvested at the time of surgical resection. Samples, named Glio9, Glio11, and Glio14, originated from a patient with recurrent disease (Glio9) and from treatment naïve patients (Glio11 and Glio14). Stem-like cell lines were generated as previously described [49,50]. Briefly, tumors were mechanically and enzymatically dissociated, red blood cells were lysed (Red Cell Lysis Buffer, Sigma-Aldrich, St. Louis, MO, USA), and then the single cell fraction was passed through a 40 µm filter to remove undigested particulates and tumor connective tissue. Cells were then plated in stem cell media: a 3:1 ratio of Dulbecco’s Modified Eagle Medium (DMEM) and F12 (Gibco, Carlsbad, CA, USA) supplemented with 2% Gem21 NeuroPlex Serum Free Supplement without Vitamin A (Gemini Bioscience, Sacramento, CA, USA), 20 ng/mL of human epidermal growth factor and human basic fibroblast growth factor, and 1% penicillin and streptomycin. Neurospheres were grown in a humidified incubator kept at 37 °C and 5% CO_2_. Neurospheres were passaged once spheroid diameter reached ~100 µm using 1 mL Accutase (StemCell Technologies, Cambridge, MA, USA) with a 5 min incubation at 37 °C. All cell lines were routinely tested for mycoplasma using LookOut mycoplasma PCR detection kit (Sigma Aldrich) according to manufacturer’s instructions. Confirmation of the stem cell phenotypes of Glio9, Glio11, and Glio14 was performed via immunofluorescence staining for various stem markers and has been previously published [50].

The compounds used to potentiate ER stress and rescue cells, 2-deoxy-D-glucose (2-DG) and mannose, respectively, were obtained from Sigma-Aldrich and were reconstituted in phosphate buffered solution to generate stock concentrations. Autophagy inhibitor chloroquine was also obtained from Sigma-Aldrich and reconstituted in water to generate stock solution.

### 4.2. Radiation Source

Cells were irradiated using the RS-2000 Biological Irradiator (RadSource, Buford, GA, USA) at various dosages per equipment specifications. For dose-dependent radiation effects on GSC viability without addition of 2-DG, 2 Gray (Gy) to 20 Gy was administered, and 48 h post treatment, trypan blue was used to differentiate alive versus dead cells. For all transmission electron microscopy experiments, combination-therapy viability experiments, and western blot experiments, a single-time dose of 8 Gy was utilized.

### 4.3. Viability Assay

Viability was determined using the CellTiter 96^®^ AQueous One Solution Cell Proliferation (MTS) Assay (Promega, Madison, WI, USA). Neurospheres were dissociated, and single cells were seeded into 96-well plates at a density of 1.0 × 10^4^ cells per well in 100 µL of a modified neurosphere culture media containing 5% FBS and subsequently treated [50]. Following treatment, MTS reagent (20 µL per 100 µL media) was added to each well and incubated for 1–4 h. Optical density was measured at 490 nm using a Synergy HT plate reader (BioTek, Winooski, VT, USA). To determine viability effects of radiation ± 2-DG, GSCs were treated with or without 8 Gy radiation and increasing doses of 2-DG (0–2 mM) for 72 h. Data is represented as the average of 3 separate experiments in which viability is calculated as percent of non-treated (NT). The IC_50_, or the concentration of drug at which 50% of cells was non-viable, was calculated from a composite of at least three experiments.

### 4.4. Lactate Dehydrogenase Assay

Cytotoxicity was determined by measuring the lactate dehydrogenase (LDH) activity released from damaged cells using the Cytotoxicity Detection Kit obtained from Roche Applied Science (Mannheim, Germany) as per manufacturer’s instructions. Percent cytotoxicity was calculated using the following formula, in which the low control (minimal LDH release) was untreated cells and the high control (maximal release) was lysed cells. GSCs were treated with or without 8 Gy radiation and increasing doses of 2-DG (0–2 mM) and LDH release determined 48 h later:

$$Cytotoxicity\;\%=\frac{\mathrm{experimental}\;\mathrm{value}-\mathrm{low}\;\mathrm{control}}{\mathrm{high}\;\mathrm{control}-\mathrm{low}\;\mathrm{control}}$$

### 4.5. Transmission Electron Microscopy (TEM)

Prior to treatment, the neurospheres were first passed through a 40 µm filter (Falcon, Thermo Fisher Scientific, Waltham, MA, USA) to remove any single cells or debris then all the neurospheres greater than 40 µm were passed through a 100 µm to obtain a relative homogeneous population of neuropheres (ranging from 40–100 µm). Following treatment, the neurospheres were fixed in neutral buffered 2.5% glutaraldehyde at 25 °C. The specimens were post-fixed in 1% osmium tetroxide (OsO_4_) for 10 min, dehydrated using a graded ethanol series, en bloc stained with 2% uranyl acetate in 50% ethanol for 30 min, and embedded in Spurr’s epoxy resin. Semi-thin (1 μm) and ultra-thin (<90 nm) sections were cut using a Diatome 3-millimeter diamond knife on the EM UC6 ultramicrotome (Leica Microsystems Inc., Buffalo Groove, IL, USA). Semi-thin sections were stained with toluidine blue and examined using a BX60 light microscope (Olympus, Waltham, MA, USA) equipped with a digital camera (Olympus DP71). All ultra-thin sections were stained using lead citrate to be viewed under TEM with a 1400 EM (Jeol, Peabody, MA, USA) at 80 kV.

ER luminal diameter was analyzed using ImageJ software (Version 1.52e, NIH, Bethesda, MD, USA) on five electron micrographs taken at 10,000× magnification from two independent studies, while autophagy was determined by manual count. Autophagic vesicle were defined as a membrane bound vesicle containing either intact or degraded cytoplasmic material or organelles [51].

### 4.6. Western Blot

Protein extraction and western blot analysis were performed as previously described [52]. Anti-Beclin-1, anti-cleaved caspase 7, anti-CHOP, anti-GRP78, anti-phospho-H2AX, anti-LC3, anti-PARP, and anti-p62 were obtained from Cell Signaling Technology (Danvers, MA, USA). Anti-GRP94 was obtained from Enzo Life Sciences (Farmingdale, NY, USA). Anti-α-tubulin was obtained from Abcam (Eugene, OR, USA).

### 4.7. The Cancer Genome Atlas (TCGA) Data Analysis

Genomic data on GBM patients from TCGA was analyzed using an open-access brain tumor database, GlioVis (gliovis.bioinfo.cnio.es) [53]. A total of 528 GBM patient samples and 10 non-tumor patient samples were included. Data was limited to primary tumors with any 0-6-methylguanine-DNA methyltransferase (MGMT) status. Clinical survival data for high versus low expression—75th percentile versus (vs.) 25th percentile—of UPR genes (*HSPA5* (encodes GRP78) and *HSP90B1* (encodes GRP94)) and autophagy genes (*MAP1LC3B* (encodes LC3), *BECN1* (encodes Beclin1), *SQSTM1* (encodes p62), *ATG5* (encodes ATG5), *ATG7* (encodes ATG7), and *ATG12* (encodes ATG12) was reported.

### 4.8. Statistical Analysis

Significance was determined via Mann-Whitney test for all pairwise comparisons of different treatments that were tested, ER luminal diameters, and autophagosomes per cell calculations using GraphPad Prism software (Version 6.0, La Jolla, CA, USA). GlioVis data was analyzed using a pairwise *t*-test. The results are presented as mean ± standard error mean (SEM). Significance was set at *p* < 0.05.

## 5. Conclusions

Our results indicate GSC radioresistance is, in part, achieved by overexpression and overactivation of ER stress-related pathways. Additionally, high expression levels of UPR-related genes, but not autophagy-related genes, are correlated with unfavorable patient survival. However, GSCs exhibit variable expression of UPR-related proteins like GRP78 and GRP94, indicating heterogeneity to UPR activation. Loss of GSC viability can be achieved via potentiation of UPR using multiple ER-stressors—such as radiation and the glycolytic inhibitor, 2-DG—particularly when baseline GRP78 levels are low. There may be a clinical role in pathological classification of GBM based on UPR-related proteins to determine which patients would benefit from ER stress inducing therapies; however, further investigation would first be required.

## Figures and Tables

**Table 1:** Measurements of ER diameter (microns) and autophagic vesicles per cell in Glioblastoma stem cell (GSCs) treated with 8 Gy radiation. Mann-Whitney test.

Parameter	NT	Rad (8 Gy)	*p*-Value
**ER Diameter (µm)**			
Glio9	0.055 ± 0.002 µm	0.075 ± 0.005 µm	*p* < 0.001
Glio11	0.049 ± 0.002 µm	0.086 ± 0.003 µm	*p* < 0.0001
Glio14	0.048 ± 0.001 µm	0.154 ± 0.008 µm	*p* < 0.0001
**AV per Cell**			
Glio9	0.65 ± 0.11	1.11 ± 0.08	*p* < 0.01
Glio11	0.53 ± 0.16	0.58 ± 0.17	ns
Glio14	0.25 ± 0.10	1.18 ± 0.17	*p* <0.01

Abbreviations: AV, autophagic vesicles; ER, endoplasmic reticulum; Gy, gray; ns, not significant; NT, non-treated; Rad, radiation; µm, microns.

**Table 2:** ER diameter measurements in Glio14 treated with increasing doses of 2-DG and 8 Gy radiation.

2-DG Dose	NT	Rad (8 Gy)
0.0 mM	0.045 ± 0.008 µm	0.056 ± 0.001 µm
0.5 mM	0.062 ± 0.0023 µm	0.089 ± 0.01 µm
2.0 mM	0.060 ± 0.0026 µm	0.157 ± 0.012 µm

Abbreviations: 2-DG; 2-deoxyglucose; Gy, gray; NT, non-treated; Rad, radiation; µm, micron.

## Supplementary Materials

The following are available online at http://www.mdpi.com/2072-6694/11/2/159/s1, Figure S1: GSCs treated with increasing doses of the autophagic inhibitor, chloroquine, and exposed to 8 Gy radiation, Figure S2: Kaplan-Meier curves displaying survival data from the GlioVis portal regarding autophagy related genes.
