# Supplementary material for: Targeting Glioblastoma Stem Cells with 2-Deoxy-D-Glucose (2-DG) Potentiates Radiation-Induced Unfolded Protein Response (UPR)
Source: Cancers (Basel). 2019 Jan 31;11(2):159. doi: 10.3390/cancers11020159 (PMC6406669; doi:10.3390/cancers11020159)
Supplement: Supplementary file 1 [file cancers-11-00159-s001.pdf]

Supplementary Materials: Targeting Glioblastoma Stem Cells Via Potentiation of Radiation-Induced ER Stress Using 2-Deoxy-D-Glucose

Sumedh S. Shah, Gregor A. Rodriguez, Alexis Musick, Winston M. Walters, Nicolas de Cordoba, Eric Barbarite, Megan M. Marlow, Brian Marples, Jeffrey S. Prince, Ricardo J. Komotar, Steven Vanni and Regina M. Graham

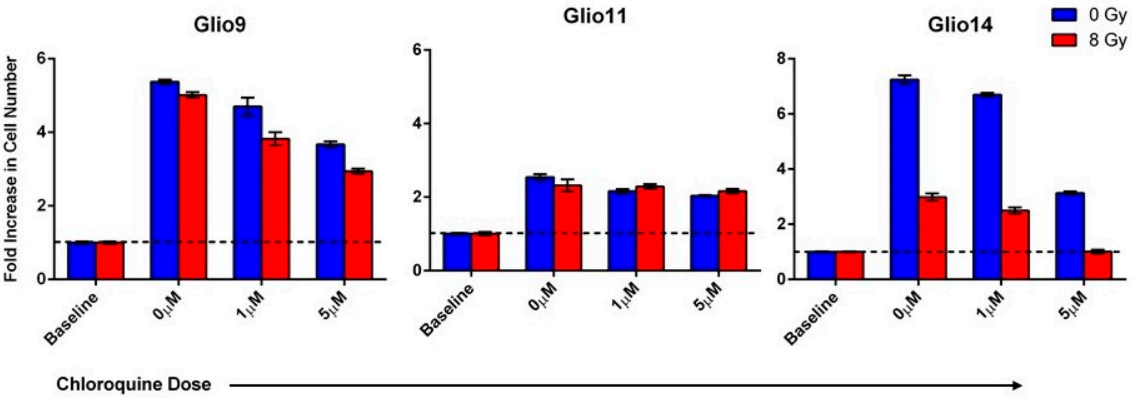

Figure S1. GSCs treated with increasing doses of the autophagic inhibitor, chloroquine, and exposed to 8 Gy radiation. Fold increase in cell number calculated using MTS assay after 72 h. Glio14 exhibit dose-dependent loss of viability when treated with chloroquine, an effect potentiated by treatment with radiation, while Glio9 exhibited moderately decreased cell viability with combination therapy. Viability of Glio11, the only GSC line without increased autophagosome formation, is not affected by radiation plus chloroquine. Results are representative of at least three experiments and displayed as mean ± SEM.

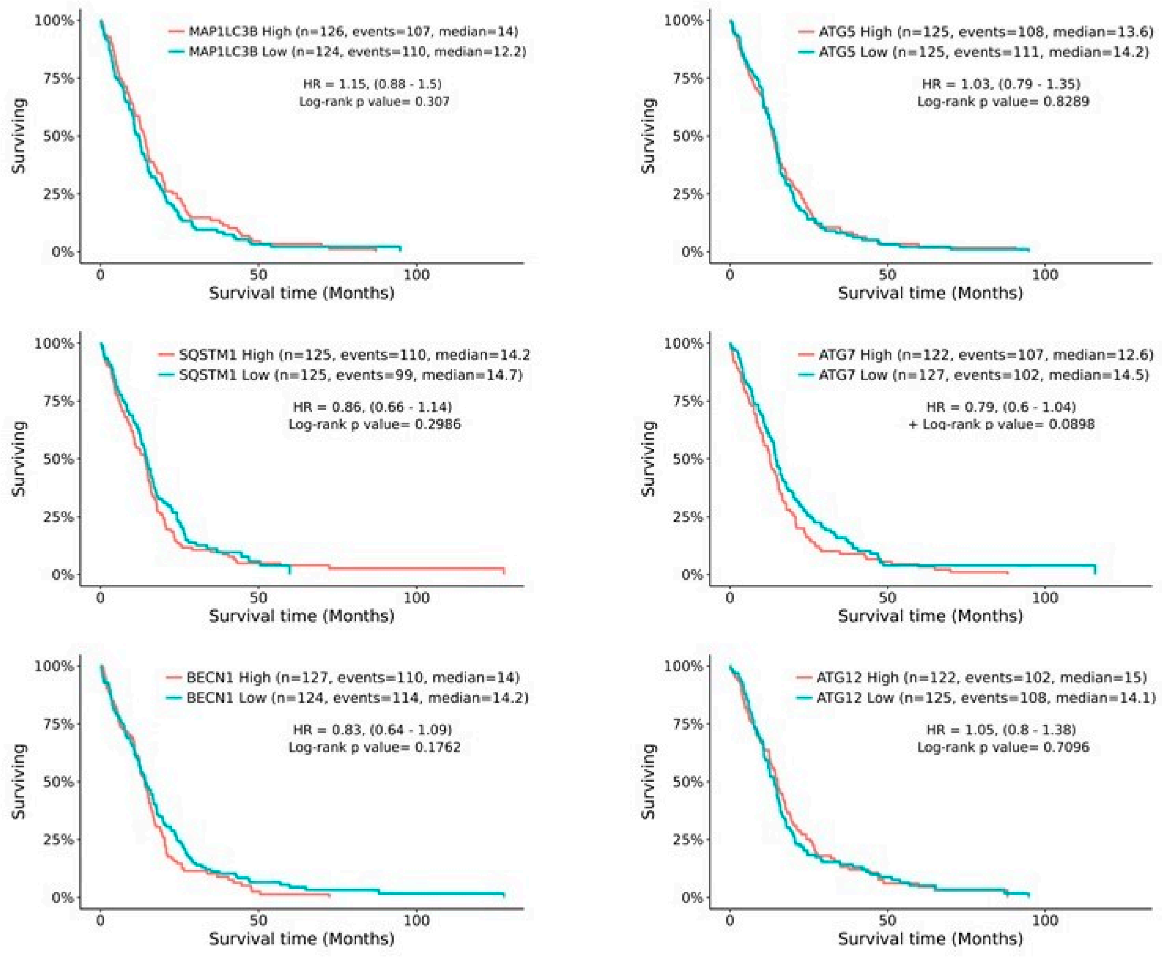

Figure S2. Kaplan-Meier curves displaying survival data from the GlioVis portal regarding autophagy related genes. From top left to bottom right: genes encoding LC3, Beclin1, p62, ATG5, ATG7, and ATG12 were not correlated with patient survival. Results reported as hazard ratio (95% confidence interval). Log-rank test. Events = number of patients who died.

© 2019 by the authors. Submitted for possible open access publication under the terms and conditions of the Creative Commons Attribution (CC BY) license (http://creativecommons.org/licenses/by/4.0/).
